# Increase of free Mg^2+ ^in the skeletal muscle of chronic fatigue syndrome patients

**DOI:** 10.1186/1476-5918-5-1

**Published:** 2006-01-11

**Authors:** Kevin K McCully, Emil Malucelli, Stefano Iotti

**Affiliations:** 1 Department of Kinesiology, University of Georgia, Athens GA 30602; 2 Dipartimento di Medicina Clinica e Biotecnologia Applicata, Università di Bologna, 40138 Bologna, Italy

## Abstract

In a previous study we evaluated muscle blood flow and muscle metabolism in patients diagnosed with chronic fatigue syndrome (CFS). To better understand muscle metabolism in CFS, we re-evaluated our data to calculate free Magnesium levels in skeletal muscle. Magnesium is an essential cofactor in a number of cell processes. A total of 20 CFS patients and 11 controls were evaluated. Phosphorus magnetic resonance spectroscopy from the medial gastrocnemius muscle was used to calculate free Mg^2+ ^from the concentrations and chemical shifts of Pi, PCr, and beta ATP peaks. CFS patients had higher magnesium levels in their muscles relative to controls (0.47 + 0.07 vs 0.36 + 0.06 mM, P < 0.01), although there was no difference in the rate of phosphocreatine recovery in these subjects, as reported earlier. This finding was not associated with abnormal oxidative metabolism as measured by the rate of recovery of phosphocreatine after exercise. In summary, calculation of free Mg^2+ ^levels from previous data showed CFS patients had higher resting free Mg^2+ ^levels compared to sedentary controls.

## Muscle Mg^2+^ in CFS

In a previous study we evaluated muscle blood flow and muscle metabolism in patients diagnosed with chronic fatigue syndrome (CFS) [1]. In this study as well as others [2-4], it has not been clear whether muscle metabolism is abnormal in CFS. To better understand muscle metabolism in CFS, we re-evaluated our data to calculate free Magnesium levels in skeletal muscle.

Magnesium is an essential cofactor in a number of cell processes. Magnesium ions influence the equilibria of many reactions involved in cellular bioenergetics interacting with phosphorylated molecules and interfering with the kinetics of ion transport across plasma membranes. Most ATP in cells is bound to Mg^2+ ^since MgATP^2- ^is the active species in enzyme binding and the energy producing form in active transport and muscular contraction [5]. Therefore, any alteration in free Mg^2+ ^could have significant consequences in muscle metabolism. Evidence for changes in intracellular magnesium has been reported in a number of diseases [6-9] and a previous study showed a free Mg^2+ ^impairment in the skeletal muscle of patients with disorders of glycolytic metabolism [10].

Resting free magnesium levels were calculated from previously collected data [1]. The subjects were CFS patients diagnosed based on the case definition of CFS were compared to inactive control subjects [11]. Magnetic resonance spectroscopy was used to obtain phosphorus metabolites from the medial gastrocnemius muscle with a 2.1 Tesla magnet. The area of each metabolite signal was fitted to a Lorentzian line shape using a time-domain fitting program AMARES/JMRUI [12], the PCr and Pi concentration were calculated by assuming a normal ATP concentration of 8 mM. The cytosolic pH and [Mg^2+^] were calculated from the chemical shift of Pi and β-ATP respectively, both measured from the resonance of PCr, using an equation which takes into account the mutual influence between pH and [Mg^2+^] [12].

**Figure 1:**
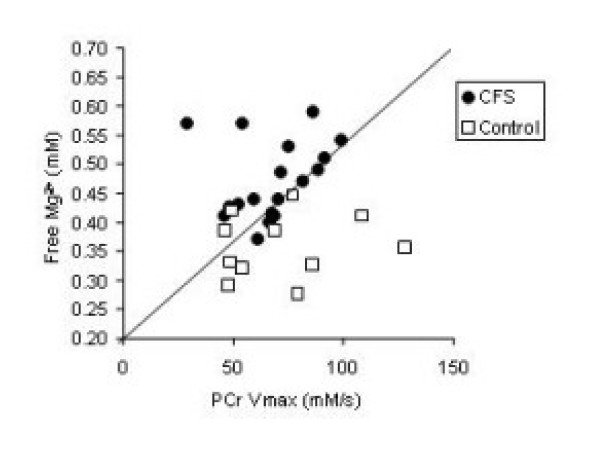
Comparison of free magnesium and oxidative metabolism measured by PCr recovery in the legs of CFS and control subjects. While PCr recovery values were not different between groups, the CFS patients had significantly higher values of free magnesium.

A total of 20 CFS patients and 11 controls were evaluated. Values from both legs were averaged. CFS patients had a significantly higher level of free Magnesium compared to controls (Table 1, P < 0.01). However, there were no differences in resting PCr levels and pH. As reported earlier, there were no differences in oxidative metabolism measured as the rate of phosphocreatine recovery in these subjects. Figure 1 clearly shows that despite having normal PCr recovery values, the CFS patients had higher free magnesium.

**Table 1:** Resting metabolite values for control and CFS subjects. Values are means and SD.

		PCr (mM)	pH	Mg^2+ ^(mM)
**Controls**	Mean	34.88	6.94	0.36
N = 11	SD	3.19	0.02	0.05
**CFS**	Mean	34.72	6.93	0.47
N = 20	SD	3.18	0.03	0.07

Our analysis showed CFS patients to have higher magnesium levels in their muscles relative to controls, which is a unique observation. This was true for the population as a whole, although there was overlap in the two populations. This finding was not associated with abnormal oxidative metabolism as measured by the rate of recovery of phosphocreatine after exercise. It is not clear what the implications of having higher free magnesium levels are, since the alteration in free Mg^2+ ^found in other pathologies usually refers to a lower concentration [6]. Previous studies have outlined that free magnesium is related to intracellular pH, but in our study there was no difference in pH between CFS and controls, and there was no relationship between resting free magnesium and resting pH. It is known that a decrease in ATP content causes an increase of Mg^2+ ^concentration, nevertheless the resting ATP level was normal in the skeletal muscle of our patients.

In summary, calculation of free Mg^2+ ^levels from previous data showed CFS patients had higher resting free Mg^2+ ^levels compared to sedentary controls. While this observation was not linked to abnormal muscle oxidative metabolism, further studies are needed to test whether free magnesium levels may indicated some as yet unexplained aspect of CFS.
